# Monitoring rhinoceroses in Namibia’s private custodianship properties

**DOI:** 10.7717/peerj.9670

**Published:** 2020-08-14

**Authors:** Zoe C. Jewell, Sky Alibhai, Peter R. Law, Kenneth Uiseb, Stephen Lee

**Affiliations:** 1 Nicholas School of the Environment, Duke University, Durham, NC, United States of America; 2 JMP Division, SAS, Cary, NC, United States of America; 3 WildTrack, Durham, NC, United States of America; 4 African Centre for Conservation Ecology, Nelson Mandela University, Port Elizabeth, South Africa; 5 Directorate of Scientific Services, The Ministry of Environment and Tourism, Windhoek, Namibia; 6 US Army Research Office, Cary, NC, United States of America

**Keywords:** Rhinoceros, Population monitoring, Namibia, Non-invasive, Footprints, Tracking, Custodianships, Traditional ecological knowledge, Heel patterns, Footprint identification technique

## Abstract

Routinely censusing rhinoceros’ populations is central to their conservation and protection from illegal killing. In Namibia, both white (*Ceratotherium simum*) and black (*Diceros bicornis*) rhinoceros occur on private land, in the latter case under a custodianship program of the Namibian Ministry of Environment and Tourism (MET). Black rhinoceros custodian landowners are responsible for the protection of the rhinoceroses on their land and are required to report regularly to the MET. Monitoring imposes a financial burden on custodians yet many of the techniques used involve expensive monitoring techniques that include the need for aerial support and/or animal instrumentation. During May and June 2018, WildTrack undertook a pilot study to census black and white rhinoceros on three private custodianship properties in Namibia. We tested three footprint identification methods for obtaining estimates of rhinoceros populations in an effort to provide less costly alternative monitoring options to rhinoceros custodians. The first was a full monitoring protocol with two components: (a) tracking each individual animal and matching them to their footprints, (b) identifying those individuals from the heel lines on the prints. The second method used simple visual heel line identification ex-situ, and the third method used just an objective footprint identification technique. These methods offer different options of fieldwork labour and cost and were designed to offer monitoring options to custodians that provided information about rhinoceros movement and location, with minimal disturbance to the rhinoceros, and best matched their human and economic resources. In this study, we describe the three methods and report the results of the pilot study to compare and evaluate their utility for rhinoceros monitoring. The first method successfully matched each trail photographed to a known rhinoceros at each site. When the other two methods disagreed with the first, they did so by failing to match single trails to a known rhinoceros, thereby creating fictitious identities consisting of a single trail. This failure occurred twice in one application, but otherwise at most once. We expect this failure can be eliminated through more stringent criteria for collecting photographs of footprints. We also briefly compare the use of footprint monitoring with other commonly used monitoring techniques. On this basis, landowners hosting rhinoceros can evaluate which method best suits their needs and resources.

## Introduction

The IUCN lists the black rhinoceros (hereafter, ‘rhino’) (*Diceros bicornis*) as critically endangered, with a global population estimate in 2016 at between 5,053–5,467 black rhinos (Knight, 2016).

The majority (96.1%) of the population is now confined to Namibia, South Africa, Zimbabwe, and Kenya (Emslie, 2012; Moodley et al., 2017). Namibia is estimated to host about 28% of the population and is the last stronghold of the southwestern subspecies *Diceros bicornis bicornis* (Save the Rhino, 2019a). Due to low human density in Namibia, the government has been able to identify usable black rhino habitat outside National Parks. In 1993, attempting to re-establish viable sub-populations in these areas, Namibia established a Black Rhino Custodian Program, offering private landowners the opportunity to host and protect black rhinos for the government. The aim was to ‘seed’ populations for growth, spread the risk of poaching, and diversify tourism benefit (Göttert et al., 2010). After 25 years this program is considered a great success as an innovative approach to wildlife conservation. An estimated 15–20% of Namibian black rhinos are hosted by custodians who each sign a contract with the government to become responsible for the monitoring and protection of the rhinos in their care. Namibia’s custodianship program has become a global model for devolved wildlife management based on sustainable use.

Monitoring by custodians is mandated by the MET for two main purposes: to obtain reliable population demographic data to assess breeding success and population growth rates at each site; and to be able to rapidly act on any poaching.

Routine monitoring to obtain reliable data on numbers and distribution of rhinos is central to their population recovery and protection from poachers. Black rhino monitoring methods have ranged from classic field observations (Goddard, 1967), to ear and horn notching (Ngene et al., 2011), to VHF and GPS collars, horn implants and GPS anklets (Alibhai & Jewell, 2001a; Jewell, 2013; Linklater et al., 2010; Seidel et al., 2019). Schwabe et al. (2015) reported on the use of camera-traps to monitor black rhinos post-release into a new area, finding that stationary traps around waterholes performed well, but temporary cameras situated in less open terrain did not. In the authors’ experience (Alibhai & Jewell, 1997) individual rhinos imaged at night, when they come to drink, tend to be difficult to identify unless they clearly show ear notches or other distinguishing features. Footprint identification has traditionally been the preserve of expert indigenous trackers (Stander et al., 1997) and was widely used in India for monitoring tiger populations as the ‘pugmark’ technique. This was a simple and subjective visual assessment of footprint identification, but one that had the benefit of being applicable over a wide area of Indian forest land by deploying forestry employees who had expertise in tracking. However, it was generally abandoned in the late 1990’s in favour of camera-trap monitoring that was perceived to be more scientific (Karanth & Nichols, 1998). This is in contrast to African studies that showed scientifically robust individual identifications can be achieved by indigenous trackers of footprints (Stander et al., 1997) and more recently also the potential benefits of linking these skills to modern technology (Liebenberg et al., 2017). Since their introduction, challenges have also emerged in the deployment of camera-traps (Burton et al., 2015; Apps & McNutt, 2018). All the methods described above have been available to monitor white rhinos (Conway & Goodman, 1989; Ferreira, Botha & Emmett, 2012; Ferreira et al., 2015; Rees, 2018).

The authors’ interest in identifying rhinos from footprints came from their finding that female black rhinos that were immobilized for repeated instrument fitting and (one-off) ear-notching exhibited significantly longer inter-calving intervals (Alibhai, Jewell & Towindo, 2001b). After working with trackers in Zimbabwe in the 1990s, the authors developed a Footprint Identification Technique (FIT) as a non-invasive method of monitoring, initially for black rhinos (Jewell, Alibhai & Law, 2001) and subsequently extended to a variety of species (Gu et al., 2014; Jewell et al., 2016; Alibhai, Jewell & Evans, 2017; Li et al., 2018; Moreira et al., 2018). FIT uses a statistical model based on footprint morphometrics that requires footprints with clear outlines. The availability of these well-defined prints depends on many factors including wind, rain and suitability of substrate. FIT has been successfully implemented to census the white rhino population at Otjiwa game ranch in Namibia on three occasions (1999, 2002, 2004; Alibhai, Jewell & Law, 2008; Law, Jewell & Alibhai, 2013) and trialed against attempts to visually identify black rhinos using photography of nocturnal visitations at waterpoints near full moon (Alibhai & Jewell, 1997).

Until the surge in poaching in southern Africa beginning about 2008, the southern species (*Ceratotherium simum simum*) of white rhino was considered secure. Nearly 9000 African rhinos were poached in the decade ending 2018 (Save the Rhino, 2019b), the majority being white rhinos, threatening the viability of the white-rhino population (Ferreira et al., 2015; Ferreira et al., 2018). In Namibia, white rhinos occur predominantly on private land and are the exclusive responsibility of the landowner. Security of white rhinos has therefore become of greater concern, both on public and private land.

Since 2014, intensive rhino poaching for horn has expanded into Namibia from South Africa (Merem et al., 2018). The onslaught has seen at least 160 Namibian black rhinos killed in the last two years of available records, 2015–2016 (Poaching Facts, 2019). To combat poaching, Namibia has engaged both police and the military in key locations and undertaken intensive rhino dehorning, on the basis that dehorned rhinos are less attractive targets for poachers.

The rapid removal of rhinos by poaching also harms ecosystem health. As megaherbivores, rhinos are considered important ecological engineers (Waldram, Bond & Stock, 2008). Black rhinos have a range associated with high biodiversity due to their selective browsing (Muya & Oguge, 2000) and both black and white rhino are currently identified by conservationists as ‘umbrella species’, since protecting their range also conserves many other species (Roberge & Angelstam, 2004; Cromsigt & Te Beest, 2014). Therefore, saving rhinos from poaching preserves ecosystem health and biodiversity.

The loss of rhinos and the costs of combating poaching have caused financial strain on the Namibian people and government. Custodians may also lose revenue from tourists who want to observe wild rhinos with intact horns (Wright, Cundill & Biggs, 2018). Rhino protection and dehorning programmes are very costly, and rhinos must be dehorned every three years as their horns regrow. In 2017 Namibia dehorned 451 black rhinos at the significant cost of over N$14.5 million (about US$980,345) (The Namibian, 2019).

Namibia, as a black rhino stronghold, urgently needs a cost-effective, and thus sustainable, long-term strategy to monitor their rhinos. While funds have been allocated to government conservation land, private custodians are expected to fund and undertake their own monitoring. Rhino populations in private custodianships are potentially at high risk. The illegal poaching of rhinos pushes this species closer to extinction, could damage ecosystems and see a reduction in biodiversity, with loss of tourism revenue to the Namibian custodians and government. Effective monitoring to inform conservation strategy is key to reversing this decline and preserving rhinos into the future.

WildTrack, a 501(c)3 non-profit registered in North Carolina, has been working with the Namibian Ministry of Environment and Tourism (MET) since 1997 to monitor both black and white rhino in National Parks and on private conservancies. On the basis of our prior experience censusing and monitoring rhino populations in Zimbabwe and Namibia, we developed three methods for censusing and monitoring rhino that exploit rhino footprints as the primary data. One of these methods is WildTrack’s FIT, which our prior studies have focused on testing in the field (Jewell, Alibhai & Law, 2001; Alibhai, Jewell & Law, 2008). This study compares the performance and costs of the three different protocols for using footprints to monitor rhinos. All three methods were tested at each of three different black rhino custodian properties, one of which also included a white rhino population. We compare the results of the three techniques and point out the pros and cons of each method. We also briefly compare footprint monitoring to other commonly used rhino monitoring techniques to aid custodians and others involved in rhino monitoring to evaluate which approach best suits their needs and resources.

## Materials and Methods

### Permissions statement

The authors obtained verbal and written permission to conduct this study from the landowners and the Ministry of Environment and Tourism of Namibia.

### Terminology

Census: A complete count, or estimate, of individuals in a population

Monitor: To follow the demographics and distribution of a population over time, by repeated censuses or other assessments conducted at regular intervals

Footprint: An impression in the substrate made by the foot.

Trail: An unbroken series of footprints made by one individual animal.

### Census techniques

This pilot study was conducted at three custodianship properties in Namibia in May-June 2018; sites A, B and C. Custodian sites were chosen by the MET as those that performed regular monitoring and were presumed to have reliable estimates of rhino numbers. Sites A and B were predominantly dry bushveld and Site C was mixed grassland and dry bushveld. Site locations are not disclosed to maintain rhino security. Each site hosted a small black rhino population and C also hosted white rhino. Custodian estimates of rhino populations were obtained prior to the survey and are reported in Table 1. While each custodian was confident that they knew how many rhinos they had on their property from anti-poaching and routine surveillance patrols, we regarded their estimates as a baseline to be tested by our Method One. Our further aim was to test the consistency of the three methods relative to each other and the Custodian’s estimate and compare the utilities of the three different census techniques, all based on footprint identification.

**Table 1:** Estimated black (B) and white (W) rhino numbers at three different sites, using the three different census methods of Full Census (FIDP, method 1), Heel Pattern (HP, method 2) and Footprint Identification Technique (FIT, method 3), compared with custodian estimates. Each method takes the pool of trails employed in its analysis and clusters them into groups, with trails within a group classified as like and trails in different groups classified as unlike. The resulting number of groups provides the estimate of the number of individuals. The matching of trails is illustrated in Fig. 2. Note that the recently born calf at Site A is excluded from this table and Table S1. It was discovered by Method 1. We did not photograph the footprints because the impressions had little detail other than size. Size alone, however, did distinguish it so its identity was in effect recognized by all three methods.

	Site A	Site B	Site C
Full Census (FIDP)	4(B)	8(B)	17(W); 6(B)
Heel Pattern (HP)	5(B)	9(B)	17(W); 6(B)
Footprint Identification Technique (FIT)	5(B)	8(B)	18(W); 6(B)
Custodian estimate	4 (B)	8(B)	17(W); 6(B)

Sites A and C were commercial hunting farms, A on mostly flat sandy terrain while C had flat sandy, largely grassed terrain transitioning to rocky undulating bushveld terrain. Site B was a recreational tourism site on flat sandy terrain. At each site, rhinos were monitored regularly through anti-poaching and infrastructure repair patrols, and through observations with clients. At all three sites, roads, game tracks, and bare areas around waterpoints provided generally good substrate for tracking. There was no rain during the study period which took place in the middle of the dry season.

### The three methods

Footprints were collected by photography from a thorough search of roads, tracks, and waterholes as the common component of all three methods.

#### Method 1. The Full Identification Protocol (FIDP) consisted of two components: a visual identification of individual rhino from body patterns/ear notches and a matching of heel patterns on their footprints

Guided by the custodian trackers, the aim of FIDP was to match opportunistic visuals to fresh spoor that could be photographed and to track fresh spoor, while being photographed, to a visual of the rhino. Footprints photographed with heel pattern but not matched to a visual could be compared to those footprints already matched to a visual. Absence of a match directed subsequent field searches until a visual with matching heel pattern was found. As rhino heel lines are unique to individuals, a simple visual comparison of these lines would provide custodians with the option of ongoing monitoring of individuals from footprints. The footprint images linked to individuals were included in the total number of footprints collected for use in Method 2 (Identification by heel pattern only), and 3 (FIT analysis only). See below under Field Protocol for more detail on ‘heel line pattern’, photography protocol, and FIT.

#### Method 2. Identification by heel pattern only (HP)

**Figure 1:**
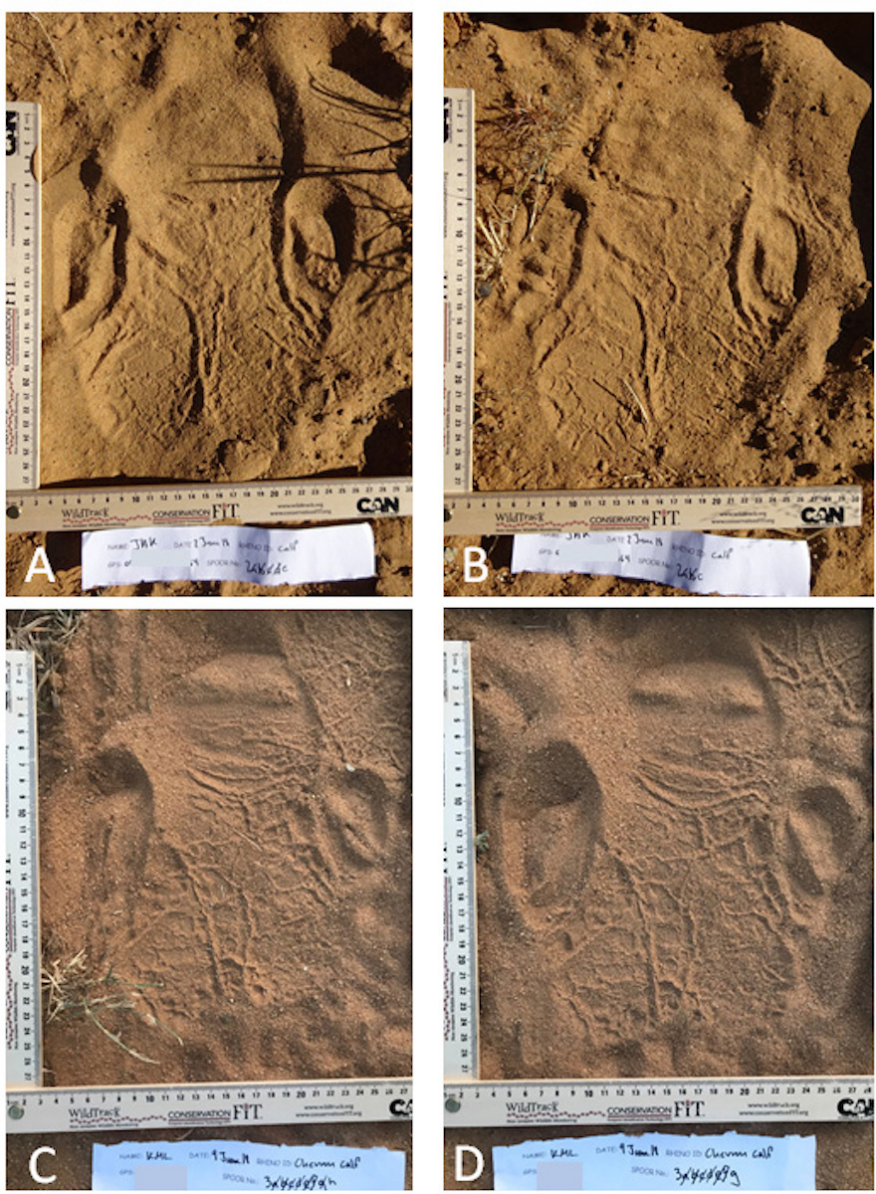
A left hind rhino footprint showing distinctive heel lines. A and B are two different footprints from the same trail made by one rhino and C and D two footprints from the same trail of a different rhino. The heel patterns can be visually matched in each pair.

This method involved a census to provide a population estimate just using the heel lines visible in all footprint images collected that were sufficiently clear. Without using any reference to known individuals, the heel lines were matched visually from the footprint images alone and processed off-site by co-author SA, who was not involved in the field work. This method is potentially quicker than Method 1, but since rhinos were not tracked to a visual, it would not match footprints to individuals. Figure 1 shows the obvious differences in heel pattern between two individuals but their similarity in different footprints of the same individual.

#### Method 3. The Footprint Identification Technique (FIT)

Heel lines were disregarded, as was any known information about the individual from whom the footprints had come. Only footprints with clear outlines of the three toes were used for this method. FIT analyses all footprints of suitable quality to estimate how many animals were represented by those footprints. As with method 2, this method does not match footprints to individuals, as is necessary for ongoing monitoring.

### Field protocol

#### Footprint quality criteria

While the chance of encountering a wild rhino is low, footprints can be much more accessible. Our experience is that footprints will last at least 24 hrs unless rain or high winds interfere. Other variables, including substrate type and other animal activity can also impact on the longevity of footprints. One of the benefits of using ‘fresh’ (<24 h old) footprints is that they inform on a rhino’s recent location and behaviour. Such footprints can be identified with experience by their clean, sharp outlines. Upon first observation, each footprint to be imaged was circled by a line in the substrate to prevent repeated sampling. The FIT protocol for rhinos requires images of multiple footprints in an unbroken trail (i.e., footprints known to be from one individual). The images are of left hind (LH) footprints that clearly show the outline of the foot and its three toes (Jewell, Alibhai & Law, 2001). Footprints that did not fit these criteria were not used in the FIT analysis. Occasionally, single images were taken of those high-quality LH footprints that exhibited a clear heel pattern when they were unambiguously associated with a visual of a rhino so that these photographs could be used as references for subsequent comparisons as explained below. Footprints that did not meet the criteria for FIT, but nonetheless contained a good heel line pattern, were also photographed for use in methods 1 and 2.

#### FIT methodology

The footprint identification technology (FIT) utilizes the traditional ecological knowledge of local trackers to find, follow and photograph animal footprints, and is based on the fact that not only do individual animals have unique foot structure, but also that each footprint itself is unique, it’s structure being dependent on substrate quality, animal gait, pace, weather conditions etc. Footprint images are collected according to a specific protocol (Jewell, Alibhai & Law, 2001 and see Fig. 1), then manually annotated with pre-determined, anatomically defined, landmark points. The images are then processed in JMP software to create ‘geometric profiles’ of the footprint, where each profile consists of a series of lengths, areas and angles on the footprint. FIT uses a model that performs a cross-validated discriminant analysis using a pairwise comparison of trails (unbroken series of footprint images) in the input dataset. Pairs of trails are simply classified as ‘self’ (from the same animal) or ‘non-self’ (from two different animals). A cluster dendrogram output provides a clear classification at the species, individual, sex or age-class level. By collecting all clear footprints regardless of substrate, FIT accounts for environmental and substrate variability and identifies an animal to the individual, age and sex levels with 95–99% accuracy. This non-invasive method allows for rapid population-level data collection (Jewell, Alibhai & Law, 2001; Jewell et al., 2016; Alibhai, Jewell & Law, 2008).

Based on prior experience, at each custodian site our objective was to collect a target of at least 30 footprints from each individual rhino, to allow for variation of substrate, walking gait, pace and other factors that might vary between individual footprints. Trails made by running rhino were not used.

#### Footprint heel pattern matching

The cracks in the pad of rhino feet, unique to each animal, can leave raised impressions in the footprint. Where the substrate texture is soft enough to deform to a clear pattern of the sole of the foot (footprint), but firm enough that this imprint is retained after the animal lifts the foot, these patterns are sufficiently distinct amongst individuals to serve for individual recognition, at least for a single field season and for not too large a population (we have previously distinguished 26 different white rhino in one season in this manner, and a total of 40 over three seasons in the same area (Alibhai, Jewell & Law, 2008). Not every LH footprint may display the entire pattern so often several images from the same trail are necessary to identify the pattern unambiguously.

Where individuals were difficult to track to a visual, we relied on matching of separate trails as follows. After each field session, the trails photographed in that session were inspected by the team leader and compared to other trails so far captured. If the trail was photographed in connection with an unambiguous visual, the identity of that trail was therefore known. If the patterns in two trails were found to match, they were considered to belong to the same individual. If one of those trails was associated to a sighting, then the identity was also known. Because a given LH footprint may only contain a partial representation of the complete pattern of the LH pad, one might, for example, have the following situation. Of three trails, one might be able to match trails 1 and 2 and 2 and 3 but not 1 and 3 directly. Nevertheless, in that situation we were able to conclude that all three trails were made by the same individual. By matching trails in this manner, we were able to create ‘trail identities’. This procedure allowed us to tally up the number of LH images constituting a ‘trail identity’ so as to assess our target of 30 images per identity.

#### Matching footprint heel-pattern trail identities to visual identities

Our objective in Method 1 was also to photograph a trail for each rhino sighting when possible and so match ‘trail identities’ to visual identities. ‘Trail identities’ not currently matched to a visual identity provided a target for visual identification. With one exception, known black rhino were unambiguously visually identifiable by ear notches or, if a calf, by association with their mother. The exception was the bull known to be the sole adult male at site A and unambiguously identifiable by sex. The white rhino were not ear notched but the rhino monitoring staff at that custodianship could reliably distinguish 17 white rhino by a combination of features when viewed in good light and at an appropriate angle/distance (horn shape, scars, sex, stage class: calf, subadult, adult) and group associations, which were stable for our short field season. In one case, however, a trio of subadults, two female and one male, although this group could be reliably identified visually, the two females within it could not be visually distinguished reliably. Records of white rhino releases, births, and deaths indicated that these 17 white rhinos constituted the white rhino population. The only possible exception would have been unobserved births resulting in individuals that remained unknown to the staff or never recognized as distinct from the other 17. Given that the potential mothers were well known, this possibility seemed remote.

With these procedures we kept records of footprint counts, ‘trail identities’ to be visually identified, and known rhino still to be associated with a ‘trail identity’. The ‘trail identities’ were only employed as part of the methodology of method 1 and were considered unknown for methods 2 and 3. For methods 2 and 3, trails collected separately in the field, whether subsequently matched by pattern or not by method 1, were treated as distinct prior to analysis in methods 2 and 3.

#### Fieldwork effort and strategies

The field team consisted of the team leader, co-author PRL, two field assistants, and a tracker provided by the custodian, in most cases charged with monitoring the custodian’s rhino population. Each day was split into a morning (from just after sunrise to about midday) and afternoon (from about 3pm until sunset) field session.

The time allotted for field work at each site was pre-determined by arrangement with each custodian, a compromise between our estimate of the minimum effort necessary and the imposition on the custodian. The evaluation of the three techniques was thus conducted under a realistic constraint on effort.

With the knowledge that all sites were fenced, and that all rhino had to visit water points at least every 2 days, water points were the primary target for footprint searches. Most water points at sites A and C were small artificial water points with only a relatively small area of bare substrate around them, which was usually trampled with spoor of ungulates of various species. Thus, water points were primarily useful for signs that rhino had visited rather than for collecting trails themselves. Whenever such signs were detected, we attempted to locate a trail where a rhino had approached or departed from the water point and then follow this trail in the pursuit of useful photographs of spoor and a possible visual. These attempts were most successful when rhinos used the roads leading to and from the water points, as tall grass was still plentiful in June off the roads. Otherwise, the roads themselves provided search patterns for trails. Site B possessed mostly larger natural water points with extensive bare areas surrounding them so that rhino spoor was not typically trampled by other ungulates. As a result, it was easier to identify trails of rhino approaching and departing from water points. Although trails on roads were still of course useful, routinely checking the water holes was an efficient protocol at site B for locating rhinos.

For white rhinos at site C, visuals could often be made of rhino grazing in the early morning in the open grasslands, and we attempted to locate the associated trails when the rhinos moved off and to track the rhinos to their resting sites, photographing acceptable footprints in the process.

We typically set out to tour water holes and roads in a section of the site each morning and could cover the site in two or three days if uninterrupted. Of course, sightings of trails and or rhinos did interrupt such routine inspections and, if promising, became the priority of the session. Afternoons either resumed where the morning left off or started a new routine tour. As time passed, we also used an adaptive protocol in focusing more attention in areas where greater rhino activity was detected or focused on locating more elusive individuals.

## Results

### Field notes

At site C, we focused first on the white rhino, beginning on 29 May 2018. By 9 June we had achieved visual identities on each of the 17 white rhinos known to the staff, sexed the two most recent calves (previously unsexed), and unambiguously photographed and matched footprints to each of these individuals. Every ‘trail identity’ established in the field was matched to a known rhino.

The black rhino at site C had been introduced recently as a group of six. We collected footprint images for black rhino at site C from June 1 through 10, focusing most of our attention on the black rhino from June 5 through 10. We obtained visuals on four of the six, identified the footprints of one other seen by a professional hunter (identified by ear notches), and had unambiguously photographed trails from these five black rhinos plus another trail we did not match to these five. These black rhinos were walking extensively along roads, still apparently exploring their new landscape. This fact allowed us to capture many images from single trails for some of these black rhinos.

Although the black rhinos were long established at site A, they did not confine their movements to small ranges, though there were parts of site A that rhinos apparently did not utilize. Between 21 and 29 June, we obtained visuals on all four known adult black rhino at site A and obtained the first visual of a recently born calf for which only spoor had previously been reported, thereby identifying its mother. We also obtained unambiguous spoor photographs for each adult rhino and all ‘trail identities’ were matched to known rhino.

Between 13 and 19 June we obtained visuals on each of the eight black rhinos known to staff at site B, obtained unambiguous footprint images for each of these individuals, and matched each ‘trail identity’ to a known rhino.

### Analysis

More than 1,500 footprints were collected from the three sites, over a five-week period. 608 photographs of white rhino footprints and 331 black rhino footprints were collected at site C over 17 days. 402 photographs of footprints of black rhino were collected at site B over 7 days and 300 at site A over 7 days. Over the entire period of five weeks, 45 separate visuals (sometimes of multiple groups of white rhinos, and for both species occasionally of a bull accompanying, or near, a female rhino) were achieved. 23 visuals resulted from tracking footprints; the remainder began as a visual from a vehicle. Footprints were analysed as described above, and grouped by individual identity, according to the three methods. The individual groupings for the animals at each site are presented in Table S1 (Site A), S2 (Site B) and S3,4,5 (Site C)

Table 1 presents, for each site, the number of rhinos estimated by each of the three different methods along with the custodian’s prior estimate of the number of their rhino. Each method takes the pool of trails employed in its analysis and clusters them into groups, with trails within a group classified as like and trails in different groups classified as unlike. The resulting number of groups provides the estimate of the number of individuals.

At all sites, Method One confirmed the Custodian estimates. At site A, methods Two (HP) and Three (FIT) each estimated an additional individual. At site B, FIT estimated the same number of rhinos as FIDP, but HP estimated one additional rhino. At site C, all three methods agreed for black rhino. For white rhino, HP gave the same estimate as FIDP while FIT estimated 18 individuals; however, with the benefit of FIDP, it is known that no trail for one of the known white rhinos proved suitable for FIT analysis so in fact FIT recognized 16 of the known white rhinos but also created two additional identities.

## Discussion

The results of this study demonstrate some of the benefits of using footprint identification to monitoring rhinos and offer a comparison of three different footprint-based approaches.

### Comparing and implementing the three different footprint methods

**Figure 2:**
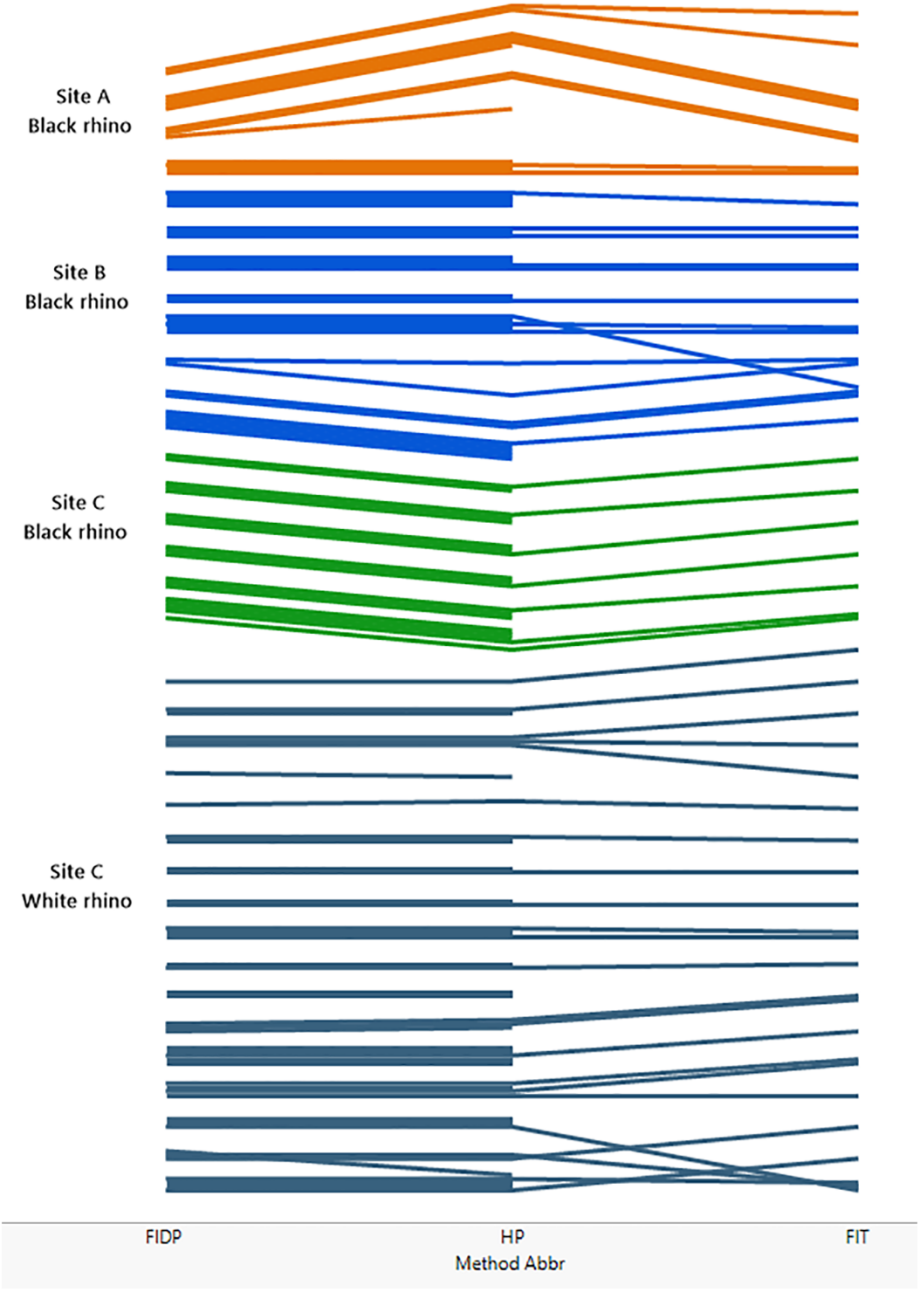
Comparing the performance of methods 1, 2 and 3 across the three sites A, B and C. The matching of trails is illustrated in figure. Each trail is represented by a coloured line, the colours coding for site and, for site C, species. At the left of the figure, lines are grouped by method 1 (FIDP), in the middle of the figure by method 2 (HP) and on the right by method 3 (FIT). Thus, if two trails are grouped together by method 1, their lines are grouped together on the left of the diagram. If those two trails are also grouped together by method two, they stay together as far as the middle of the figure, while if the trails are not in the same group per method two, their lines diverge from left to right until the middle, ending up grouped with the lines that method two groups them with. If two trails not grouped together by method one are grouped together by method two, those two lines converge, from left to right, in the middle of the figure. This process continues from left to right from the middle to the right of the figure to show the grouping by method 3 at the far right. Thus, lines that stay together across the whole diagram represent trails that are classified as like by all three methods, while lines that do not are differently classified. A line that does not extend across the entire diagram represents a trail that could be employed in one method but not another. Specifically, method 3 (FIT) is the more demanding method while methods 1 and 2 can also utilize heel patterns when the outline of the footprint is inadequate for FIT.

Figure 2 (see also Tables S1–S5) compares the performance of the three different methods across the three sites and indicates that the individual rhino groupings of trails generally agreed across all three methods at each site. In a few instances the trail identities disagreed, resulting in the groupings diverging from each other. These divergences were mostly due to low numbers of footprints in a trail. Method 3 (FIT) was the most demanding in terms of footprint clarity, and not all the trails that could be assessed by methods 1 and 2 were accessible to method 3 resulting in some groupings not extending to the far right of Fig. 2 that shows the FIT classification.

The most intensive method, FIDP, provided results that agreed with the custodian estimates and is also most informative about a population of rhinos. As the only method of the three that matches footprint images to visual confirmation of an individual, and thus provides the custodian with a reference set of images matching each individual to its footprints, it also allows custodians to continue to monitor individuals without prior knowledge of the population. On the other hand, the data required for methods 2 and 3 can be collected as part of the custodianship’s routine fieldwork, requiring only that this be extended to include photographing of footprints, an activity which in itself improves knowledge of the whereabouts and movements of rhino, and thereby the effectiveness of the routine monitoring. It would also draw attention to individuals whose footprints were not being observed once a library of footprints of all individuals is established.

That every trail photographed for the FIDP method was ultimately matched to a rhino known by a visual identity not only confirmed the Custodian estimates but provides evidence for its utility as a complete census. Accepting the resulting censuses provided by FIDP, method 2 (HP) erred at sites A and B by inferring one extra identity at each. Each of these identities consisted of a single trail not matched by HP to any other trail in the site’s database. For site A, that trail was photographed under a midday sun, and despite shading the footprint and using a reflector to attempt to draw out contrast, the photographs show little detail of the heel pattern, and less than was observed in the field. This trail was together with the trail of another rhino. We had seen and photographed two trails together two days previously and were able, with the detail observable in the field, to match these two trails to the previous ones; in particular, the trail constituting the additional identity in method 2 was recognized by the field workers as belonging to the bull at site A. At site B, the additional identity under method 2 also consisted of a single trail. Through method 1, it was known to the field workers that this trail was collected as the trail of the calf in a cow-calf pair. Each trail manifests variation in the heel pattern impression and highlights the difficulty that can be encountered with relying on heel patterns alone.

Method 3, FIT, agreed with method 1 at site B and for black rhino at site C. At site A, FIT, assigned an extra identity, which consisted of a single trail known to belong to Cow8 through tracking. For white rhino at site C, no trail for an adult female with calf designated cow4 proved acceptable for FIT analysis so this identity does not occur for FIT, but FIT did recognize the other 16 white rhino. In addition, two trails whose identities were known by FIDP were not matched to any other trail in the site C database by FIT and thus each constituted a separate identity.

Thus, each fictitious identity produced by methods 2 and 3 as judged by method 1 consisted of a single trail only, for which method 1 provided additional information through tracking and visuals to identify. In this pilot study we sometimes photographed trails that were less than ideal for either methods 2 or 3 because with supplementary field information they were useful in method 1. The fact that methods 2 and 3 never created a fictitious identity consisting of more than one trail suggests that the trails yielding the fictitious identities can be considered outliers for either methods 2 or 3 and that the precision of these methods can be improved by stricter adherence to the protocols for collecting photographs for these methods. At the same time, identities consisting of a single trail of less than ideal quality can be highlighted as suspect and deserving follow-up investigation. We also note that the incidence of this error did not increase with population size; specifically, the per capita rate decreased with population size.

Table 2 compares the three different methods in terms of speed of delivery, accuracy, and relative cost. It should be noted that method 2 does involve some subjective judgement and expertise. In this study method 2 was performed by co-author SA, who has years of experience examining rhino footprints. While demanding in its protocol for collecting footprints, FIT offers the attraction of being completely objective. As already noted, methods 2 and 3 exploit different information in the footprint and one may prove more effective on some substrates than the other.

**Table 2:** Comparing features of the three different footprint-based survey methods relative to each other in terms of speed of deployment, accuracy and relative cost.

	Speed of delivery	Accuracy	Relative cost
Full Census (FIDP)	Slower	Most accurate	More costly
Heel Pattern (HP)	Faster	Good accuracy	Least costly
Footprint Identification Technique (FIT)	Medium	Good accuracy	Medium cost

### Recommendations on the application of methods 1–3

Custodians are required to obtain regular sightings of their rhinos and report to the Ministry of Environment and Tourism. If an individual is not seen within the allocated time, an intensive search is required. To fulfill these demands, they must make a strategic choice of monitoring strategy depending on their resources including staff availability and expertise, and the budget available for monitoring. Terrain suitability and climate will also dictate the method(s) chosen. We recommend that a full ID protocol (method 1) should be undertaken at each custodianship annually if possible. In between times, and at least monthly, custodians can either use their own team or host a team trained in the protocol to do either a quick onsite survey (method 2) or take images using the FIT protocol to submit to an off-site survey (method 3). Any or all of these approaches can and should be combined and/or augmented using camera-traps and regular traditional patrols on foot or waterhole counts to ensure that all rhinos are seen regularly (see below). Clearly it is in the interests of the custodians to be self-sufficient in rhino monitoring, and the WildTrack non-profit program aims to provide training and a JMP software license without cost to all custodians who are interested in using FIT to monitor their rhinos. The training can be for data collection, or for collection and analysis, depending on custodian staff resources.

During the fieldwork, other demographic observations were made. All custodians shared some natural uncertainty about the sex of new calves and the identity of their dam. The ground survey teams were able to locate all the new calves, sex some of them from direct observation and identify their mothers. At site A, trackers occasionally attributed a rhino identity to a trail based on its location, rather than careful inspection of the pad impression. We have encountered this behavior in previous field work. New light has been shed on black-rhino recursive movement patterns (Seidel et al., 2019) that could help explain these tracker assumptions. By including tracking into the monitoring regime, movements of rhino would be better known to those charged with monitoring, leading to a greater familiarity with their recursive movements within their home range. This information not only aids in keeping tabs on rhinos but also provides useful demographic information, such as which males are spatially co-extensive with which females and whether a single bull is dominating over others.

Footprints, in the form of the three methods tested in this study, not only provide a monitoring tool but also current information such as ranging and social interactions. When the team arrived at site B, staff reported seeing footprints on their walks and were unsure of the identity of the animal that made them. Tracking and FIT identified these as from the younger bull at this location. Method 1 will provide the most information of this sort and consequently, the most accurate representation of the population.

### Comparison with other monitoring options

Although a detailed analysis of costs and other benefits is outside the remit of this paper, it is worth considering, from the perspective of a custodian, how footprint identification compares with other available techniques. Table 3 compares the performance of five different methods of monitoring. Although the costs of some monitoring techniques have been published for different species (Christie et al., 2016; Claus, 2017), absolute comparisons are very difficult to make over different sized areas, different ecosystems, times of year, providers and users. The comparison in Table 3, based on our experience of many years working in this field, gives a best estimate of relative costs and benefits of these different methods as a baseline for custodians to explore further with local variables.

There are several advantages to the use of footprints compared with other current alternatives. In terms of a cost-benefit ratio, footprint identification ranks relatively highly because it requires only minimal equipment (a camera, GPS and pencil/paper/ruler) and can engage scouts who are already on site for anti-poaching/tourist work. However, it is only effective where the substrate will hold clear footprints, where expert trackers are available, and where the weather does not compromise the integrity of the footprints. Also, our experience indicates that as an animal ages, the heel patterns and footprint morphometrics may change. In our experience of work at the same site over several years (Alibhai, Jewell & Law, 2008) this does not appear to affect the accuracy of the FIT algorithm if monitoring is conducted at least once a year. To date, the largest population we have surveyed is 40 white rhinos over three years at this same site. We anticipate that the technique would scale up reasonably well if monitoring is carried out routinely.

Where trackers are available, the use of a footprint-based method can also act to leverage their traditional ecological knowledge (TEK) and, if trainees are engaged, revive these skills for the benefit of wildlife conservation. Trackers are often employed by hunting or photographic tourism sites specifically to locate an animal, or for anti-poaching duties. However, where they are employed as part of an ongoing rhino monitoring strategy their TEK about rhino ecology and behavior can be integrated into the process, contributing to local capacity in wildlife custodianship and helping develop sustainable monitoring in the absence of external experts. Footprint monitoring techniques can thus be particularly cost-effective in leveraging scout TEK for monitoring at the same time as guiding and anti-poaching.

**Table 3:** Comparing commonly used rhino monitoring techniques in terms of their speed, accuracy, estimated relative cost and suitability for different local conditions.

Survey method	Speed	Accuracy	Relative cost	Suitable where
Footprint-based methods	Medium	High	Low	Expert trackers available and substrate suitable. Non-invasive approaches preferred.
Waterhole counts	Medium	Low	Low	Trackers not available. Substrate poor for footprints. Non-invasive approaches preferred.
Aerial counts (helicopter)	High	Medium	Highest	Terrain inaccessible. Funding not limited. Rapid results required.
Aerial counts (fixed wing)	High	Medium	Medium - High	Very large areas with sparsely distributed population. Low vegetation cover. Rapid results required.
Radio or satellite telemetry	Low	High for those animals instrumented	High	Funding not limited. Continuous monitoring possible to check instruments. Small numbers of animals.
Camera-trapping	Low	Medium	Medium	Cameras can be set up at waterholes where there is no other standing water. A small population of Rhino already have marks or distinctive ear-notches.

Footprint surveys, consisting of a tracker and photographer, can provide a reliable census figure with minimal equipment (cameras, GPS and rulers) in about a week for a typical custodianship. They also provide a means of matching each individual rhino on a property to its unique footprints, so that if patrols cover the ground regularly it can provide a cost-effective ongoing monitoring approach. If deployed effectively, and ideally in combination with other ground sensors (camera-traps, regular patrols), it is also a robust anti-poaching tool. With effective monitoring rhino populations will increase and animals disperse. Footprint monitoring need not be compromised by large population sizes, however, if geographic location, social groups and known movement patterns are included as prior knowledge to assist in the identification of more animals.

Aerial counts are more expensive but have the advantage of rapid deployment. However, rhinos are not always easy to spot, particularly with vegetation cover. The advent of UAV monitoring (Christie et al., 2016) will certainly reduce the costs of aerial monitoring once the technology is fully developed, but the challenge of aerial identification remains. The more traditional approach of water hole counts are typically undertaken by local staff during a full-moon to provide at least some lighting to aid visual identification during the hours when rhino come to drink, but which is still challenging. We compared footprint identification with waterhole visual counts in Etosha National Park (Alibhai & Jewell, 1997). Footprints clearly indicated that some animals were visiting more than one waterhole each night but these different waterhole encounters with the same rhino were usually counted as distinct rhino in the waterhole counts, resulting in overcounting. Fitting instrumentation, such as horn-implants, ankle bracelets and collars, is the most expensive method as it requires the considerable costs (and risks to both animals and humans) of immobilization, instrumentation, routine (often aerial) follow-up monitoring and often repetition of these tasks if the instrumentation fails (Alibhai & Jewell, 2001a; Alibhai, Jewell & Towindo, 2001b). It will also only ‘count’ those animals already instrumented and is therefore not a full monitoring method unless every animal is instrumented and maintained in that state.

## Conclusion

To summarize, every monitoring technique has pros and cons. We would argue that the best results are likely to be obtained by identifying the available resources at a site, particularly the availability of skilled trackers, and then deploying the combination of techniques that will provide the data required in the time available. The selection of which techniques to use can be guided by Table 3.

##  Supplemental Information

10.7717/peerj.9670/supp-1 Supplemental Information 1 Trail groupings for Site A using Methods 1,2 & 3 The grouping of trails produced by each of the three techniques for each of the three sites and two species.

10.7717/peerj.9670/supp-2 Supplemental Information 2 Trail groupings for Site B using methods 1,2 & 3 The grouping of trails produced by each of the three techniques for each of the three sites and two species.

10.7717/peerj.9670/supp-3 Supplemental Information 3 Trail groupings for Site C using method 1 The grouping of trails produced by each of the three techniques for each of the three sites and two species.

10.7717/peerj.9670/supp-4 Supplemental Information 4 Trail groupings for Site C, Method 2 The grouping of trails produced by each of the three techniques for each of the three sites and two species.

10.7717/peerj.9670/supp-5 Supplemental Information 5 Trail groupings for Site C, Method 3 The grouping of trails produced by each of the three techniques for each of the three sites and two species.

## Additional Information and Declarations

### Field Study Permissions

The following information was supplied relating to field study approvals (i.e., approving body and any reference numbers): Permission was granted by the landowners and the Ministry of Environment and Tourism of Namibia.

### Data Availability

The following information was supplied regarding data availability: Data are available at Figshare: Jewell, Z. (2019). ”Dataset for PeerJ rhino monitoring paper”. Figshare. Dataset. https://figshare.com/articles/Dataset_for_PeerJ_rhino_monitoring_paper/10248713.
