# Supplementary material for: Monitoring rhinoceroses in Namibia’s private custodianship properties
Source: PeerJ. 2020 Aug 14;8:e9670. doi: 10.7717/peerj.9670 (PMC7430304; doi:10.7717/peerj.9670)
Supplement: Supplemental Information 1 — The grouping of trails produced by each of the three techniques for each of the three sites and two species. [file peerj-08-9670-s001.docx]

**Trail identifications for Site A**

**Method 1: Rhino field ID and pattern match**

Black rhino (4)

| **Cow1**  21 JUN PRL 1  22 JUN PRL 1  22 JUN PRL 2  28 JUN PRL 1 | **Bull**  21 JUN PRL 2  23 JUN PRL 1  24 JUN PRL 1  25 JUN PRL 2 | **Cow4**  23 JUN PRL 2  25 JUN PRL 1  25 JUN PRL 3  26 JUN PRL 1 | **Cow8**  24 JUN PRL 2  28 JUN PRL 2  29 JUN PRL 1 |  |
| --- | --- | --- | --- | --- |

**Method 2: Heel pattern match**

Black rhino (5)

| 21 JUN PRL 1  22 JUN PRL 1  22 JUN PRL 2  28 JUN PRL 1 | 21 JUN PRL 2  24 JUN PRL 1  25 JUN PRL 2 | 23 JUN PRL 2  25 JUN PRL 1  25 JUN PRL 3  26 JUN PRL 1 | 24 JUN PRL 2  28 JUN PRL 2  29 JUN PRL 1 | 23 JUN PRL 1  (photos are overexposed due to midday light; recognized in field as spoor of bull) |
| --- | --- | --- | --- | --- |

**Method 3: FIT analysis**

Site A: Black rhino (5)

| 21 JUN PRL 1  22 JUN PRL 2 | 24 JUN PRL 1A  24 JUN PRL 1B  24 JUN PRL 1C  25 JUN PRL 2 | 25 JUN PRL 1  25 JUN PRL 3  26 JUN PRL 1 | 29 JUN PRL 1A  29 JUN PRL 1B  24 JUN PRL 2 | 28 JUN PRL 2  (photos taken in fading light but recognized via tracking as Cow8) |
| --- | --- | --- | --- | --- |
