# Supplementary material for: Monitoring rhinoceroses in Namibia’s private custodianship properties
Source: PeerJ. 2020 Aug 14;8:e9670. doi: 10.7717/peerj.9670 (PMC7430304; doi:10.7717/peerj.9670)
Supplement: Supplemental Information 2 — The grouping of trails produced by each of the three techniques for each of the three sites and two species. [file peerj-08-9670-s002.docx]

**Trail identifications for Site B**

**Method 1: Rhino field ID and pattern match**

Black rhino (8)

| **Bull C**:  13 JUN PRL 1  15 JUN PRL 2  16 JUN HNH 1  15 JUN MKB2  15 JUN MKB 3 | **Cow J**:  14 JUN JAK 1  16 JUN PRL 1 | **Calf J**:  14 JUN HNH 1  16 JUN MKB 1 | **Cow K**:  14 JUN MKB 1  14 JUN PRL 1  15 JUN MKB 4  18 JUN PRL 2  19 JUN MKB 1 | **Calf K**:  15 JUN HNH 1  15 JUN MKB 1 |
| --- | --- | --- | --- | --- |
| **Cow H**:  16 JUN PRL 2  16 JUN PRL 3  18 JUN PRL 1  18 JUN PRL 3 | **Calf H**:  16 JUN MKB 2  17 JUN PRL 1  18 JUN MKB 1 | **Bull H**:  14 JUN MKB 2  15 JUN PRL 1  16 JUN KML 1  17 JUN PRL 2 |  |  |

**Method 2: Heel pattern match**

Black rhino (9)

| 13 JUN PRL 1  15 JUN PRL 2  16 JUN HNH 1  15 JUN MKB2  15 JUN MKB 3 | 14 JUN JAK 1  16 JUN PRL 1 | 14 JUN HNH 1 | 14 JUN MKB 1  14 JUN PRL 1  15 JUN MKB 4  18 JUN PRL 2  19 JUN MKB 1 | 15 JUN HNH 1  15 JUN MKB 1 |
| --- | --- | --- | --- | --- |
| 16 JUN PRL 2  16 JUN PRL 3  18 JUN PRL 1  18 JUN PRL 3 | 16 JUN MKB 2  17 JUN PRL 1  18 JUN MKB 1 | 14 JUN MKB 2  15 JUN PRL 1  16 JUN KML 1  17 JUN PRL 2 | 16 JUN MKB 1 |  |

**Method 3: FIT analysis**

Black rhino (8)

| 3 JUN PRL 1  5 JUN MKB2  16 JUN HNH 1 | 14 JUN JAK 1A  14 JUN JAK 1B  16 JUN PRL 1  19 JUN MKB 1 | 14 JUN HNH 1  16 JUN MKB 1 | 14 JUN MKB 1  15 JUN MKB 4 | 15 JUN HNH 1 |
| --- | --- | --- | --- | --- |
| 16 JUN PRL 2  16 JUN PRL 3  18 JUN PRL 3 | 16 JUN MKB 2  17 JUN PRL 1  18 JUN MKB 1 | 17 JUN PRL 2 |  |  |

Suffixes (A, B….) denote sub-trails used for FIT analysis.
