# Supplementary material for: Monitoring rhinoceroses in Namibia’s private custodianship properties
Source: PeerJ. 2020 Aug 14;8:e9670. doi: 10.7717/peerj.9670 (PMC7430304; doi:10.7717/peerj.9670)
Supplement: Supplemental Information 3 — The grouping of trails produced by each of the three techniques for each of the three sites and two species. [file peerj-08-9670-s003.docx]

**Trail identifications for Site C**

**Method 1: Rhino field ID and pattern match**

White rhino (17)

| **Trio1a**:  30 MAYJAK 2  30 MAY PRL 2  31 MAY JAK 1  1 JUN KML1 | **Trio1b**:  30 MAY KML 3  31 MAY PRL 1  1 JUN KML 2 | **Trio1c**:  31 MAY KML 1  31 MAY KML 2  1 JUN KML 3 | **Trio2a**:  2 JUN KML 3  6 JUN KML 2  6 JUN PRL 2  6 JUN PRL 3 | **Trio2b**:  29 MAY JAK 1  2 JUN JAK 1  2 JUN JAK 2  6 JUN JAK 3  6 JUN KML 3 |
| --- | --- | --- | --- | --- |
| **Trio2c**:  2JunKML1  2 JUN KML 2  2 JUN PRL 1  2 JUN PRL 2 | **The Bull**:  30 MAY KML 1  30 MAY KML 2  4 JUN PRL 1 | **Cow1**:  8 JUN PRL 1  8 JUN PRL 2 | **Calf 1**:  30 MAY PRL 1  4 JUN KML 1  8 JUN JAK 1 | **Cow2**:  1 JUN JAK1  7 JUN KML 1 |
| **Calf 2**:  30 MAY JAK 1  1 JUN PRL 1 | **Cow 3**:  3 JUN PRL 2  9 JUN PRL 5 | **Calf 3**:  3 JUN PRL 1  9 JUN KML 3 | **Cow 4**:  4JUN PRL3  4 JUN PRL 4  5 JUN PRL 1 | **Calf 4**:  4 JUN JAK 1  5 JUN JAK 1  4 JUN PRL 2 |
| **Cow 5**:  29 MAY JAK 2  29 MAY PRL 1 | **Calf 5**:  29 MAY KML 1  29 MAY PRL 2 |  |  |  |

Black rhino (6)

| **Bull 11**:  2 JUN KML 4  2JunPRL3  5 JUN KML 1  6 JUN JAK 1  6 JUN KML 1  6 JUN PRL 1 | **Bull 7**:  1JunJAK2  6JunJAK2  10 JUN PRL 1 | **Bull 1**:  5 JUN PRL 2  6 JUN PRL 4  9 JUN PRL 1 | **Cow 3**:  9 JUN JAK 1  9 JUN KML 1  9 JUN KML 2 | **Cow 20**:  9 JUN PRL 2  9 JUN PRL 3  9 JUN PRL 4 |
| --- | --- | --- | --- | --- |
| **Cow10**:  5 JUN PRL 3  10 JUN PRL 2 |  |  |  |  |
