# Supplementary material for: Monitoring rhinoceroses in Namibia’s private custodianship properties
Source: PeerJ. 2020 Aug 14;8:e9670. doi: 10.7717/peerj.9670 (PMC7430304; doi:10.7717/peerj.9670)
Supplement: Supplemental Information 4 — The grouping of trails produced by each of the three techniques for each of the three sites and two species. [file peerj-08-9670-s004.docx]

**Trail identifications for Site C**

**Method 2: Heel pattern match**

White rhino (17)

| 30 MAYJAK 2  30 MAY PRL 2  31 MAY JAK 1  1 JUN KML1  1 JUN KML 2 | 30 MAY KML 3  31 MAY PRL 1 | 31 MAY KML 2  31 MAY KML 1  1 JUN KML 3 | 2 JUN KML 3  6 JUN KML 2  6 JUN PRL 2  6 JUN PRL 3 | 29 MAY JAK 1  2 JUN JAK 1  2 JUN JAK 2  6 JUN JAK 3  6 JUN KML 3 |
| --- | --- | --- | --- | --- |
| 2 JUN KML 1  2 JUN KML 2  2 JUN PRL 1  2 JUN PRL 2 | 30 MAY KML 1  30 MAY KML 2  4 JUN PRL 1 | 8 JUN PRL 1  8 JUN PRL 2 | 30 MAY PRL 1  4 JUN KML 1  8 JUN JAK 1 | 1 JUN JAK1  7 JUN KML 1 |
| 30 MAY JAK 1  1 JUN PRL 1 | 3 JUN PRL 2  9 JUN PRL 5 | 3 JUN PRL 1  9 JUN KML 3  29 MAY PRL 2 | 4 JUN PRL 4  5 JUN PRL 1 | 4 JUN JAK 1  5 JUN JAK 1  4 JUN PRL 2 |
| 29 MAY JAK 2  29 MAY PRL 1 | 29 MAY KML 1 |  |  |  |

Black rhino (6)

| 2 JUN KML 4  4 JUN PRL3  5 JUN KML 1  6 JUN JAK 1  6 JUN KML 1  6 JUN PRL 1 | 1 JUN JAK 2  6 JUN JAK 2  10 JUN PRL 1 | 5 JUN PRL 2  6 JUN PRL 4  9 JUN PRL 1 | 9 JUN JAK 1  9 JUN KML 1  9 JUN KML 2 | 9 JUN PRL 2  9 JUN PRL 3  9 JUN PRL 4 |
| --- | --- | --- | --- | --- |
| 5 JUN PRL 3  10 JUN PRL 2 |  |  |  |  |
