# Supplementary material for: Monitoring rhinoceroses in Namibia’s private custodianship properties
Source: PeerJ. 2020 Aug 14;8:e9670. doi: 10.7717/peerj.9670 (PMC7430304; doi:10.7717/peerj.9670)
Supplement: Supplemental Information 5 — The grouping of trails produced by each of the three techniques for each of the three sites and two species. [file peerj-08-9670-s005.docx]

**Trail identifications for Site C**

**Method 3: FIT analysis (**Suffixes (A, B….) denote sub-trails used for FIT analysis)

White rhino (18)

| 30 MAY JAK 2  30 MAY KML 1A  30 MAY KML 1B | 30 MAY KML 3 | 31 MAY KML 1  31 MAY PRL 1  1 JUN KML 1 | 6 JUN KML 2A  6 JUN KML 2B  6 JUN PRL 3 | 2 JUN JAK 2A  2 JUN JAK 2B  2 JUN JAK 2C |
| --- | --- | --- | --- | --- |
| 2 JUN PRL 1  2 JUN PRL 2A  2 JUN PRL 2B | 4 JUN PRL 2A  4 JUN PRL 2B | 5 JUN PRL 1  8 JUN PRL 1A  8 JUN PRL 1B | 30 MAY PRL 1  8 JUN JAK 1 | 1 JUN JAK 1A  1 JUN JAK 1B  1 JUN JAK 1C |
| 30 MAY JAK 1 | 9 JUN PRL 5A  9 JUN PRL 5B | 9 JUN KML 3A  9 JUN KML 3B |  | 4 JUN JAK 1 |
| 29 MAY PRL 1 | 29 MAY KML 1A  29 MAY KML 1B  29 MAY KML 1C  29 MAY KML 1D | 2 JUN KML 3A  2 JUN KML 3B | 5 JUN JAK 1 |  |

The third cell in row three is left blank to indicate that no trail identified as for Cow4 using Method 1 was suitable for FIT analysis (see Table S3).

Black rhino (6)

| 2 JUN KML 4  5 JUN KML 1A  5 JUN KML 1B | 10 JUN PRL 1 | 5 JUN PRL 2A  5 JUN PRL 2B | 9 JUN JAK 1A  9 JUN JAK 1B | 9 JUN PRL 4A  9 JUN PRL 4B |
| --- | --- | --- | --- | --- |
| 10 JUN PRL 2 |  |  |  |  |
